# Spray-On Colorimetric Sensors for Distinguishing the Presence of Lead Ions

**DOI:** 10.3390/chemosensors11060327

**Published:** 2023-06-02

**Authors:** Priyanka Shiveshwarkar, Justyn Jaworski

**Affiliations:** Department of Bioengineering, University of Texas at Arlington, Arlington, TX 76010, USA;

**Keywords:** sprayable sensor, polydiacetylene, lead, dipicolylamine

## Abstract

Sprayable stimuli-responsive material coatings represent a new class of detection system which can be quickly implemented to transform a surface into a color-responsive sensor. In this work, we describe a dipicolylamine-terminated diacetylene-containing amphiphile formulation for spray coating on to a simple paper substrate to yield disposable test strips that can be used to detect the presence of lead ions in solution. We find the response to be very selective to only lead ions and that the sensitivity can be modulated by altering the UV curing time after spraying. Sensitive detection to at least 0.1 mM revealed a clear color change from a blue to red phase. This represents the first demonstration of a spray-on sensor system capable of detection of lead ions in solution.

## 1. Introduction

We reveal here a spray-on sensing material which changes optical properties to stimuli (specifically lead ions) resulting in an observable color change. Such sprayable sensors could prove useful as widely deployable detection platforms based on their simple readout and ability to be applied to most surfaces. To first provide some context, detection systems that impart a change in color have been around since the advent of sensing technologies due to ease of analysis, particularly owing to a general lack of alternatives to visual confirmation at their inception over a century ago. Reliable approaches for colorimetric detection of targets thus have a long history but continue to be widely used to this day with perhaps one of the most well-known examples being that of litmus paper [1,2]. For widely deployable sensors, such straightforward visual identification of a target’s presence by a simple color change could offer a preferred method of choice, particularly due to its ability to function within a low resource setting. Even within the last decade, we still find that the most broadly used at-home systems for detection of targets molecules continue to provide a visual readout, such as within the format of lateral flow assays in pregnancy tests or COVID-19 tests [3,4]. This seemingly simple visualization signal, which allows a user to indirectly observe the presence of molecular targets with the naked eye, is based on elements within the sensing material that recognizes a given target and transduces the target-material interaction event into a change in the optical properties of that material. Depending on the technology used for the color-changing mechanism, a sensing element can exhibit distinct characteristics in its speed of response to target, detection limit, and reversibility in the optical properties. It is outside the scope of this work to describe the extensive variety of colorimetric sensing technologies, which includes plasmonic materials, liquid crystals, responsive dyes, and photonic crystals to name a few, so we direct the reader to existing reviews that provide a more comprehensive outlook of these materials [5,6]. In this work, we focus on the ability of our approach to spray-on fabrication of color-changing sensors based on a material known as polydiacetylene. This investigation of a spray-on formulation of polydiacetylene specifically for the detection of lead demonstrates that the selectivity of the chemically responsive polymeric material can be tuned to a chemical target of interest by modulating the specificity of the pendant side-chain head-group. While this concept is well-established, it has yet to have been translated to novel sprayable sensing materials. Furthermore, we reveal that by controlling the extent of polymerization of the material, the dynamic sensing range of the resulting sensors can be modified to higher or lower detection limits. These unique features allow for the extent of the color response to be both tunable for a given application based on the chemical target of interest and adaptable to reflect the concentration range of the target present. The fundamental assessment of the lead sensor outlined here, we hope, will serve as a guide for future work in polydiacetylene-based spray-on sensor formulations for other targets of interest.

**Figure 1.**
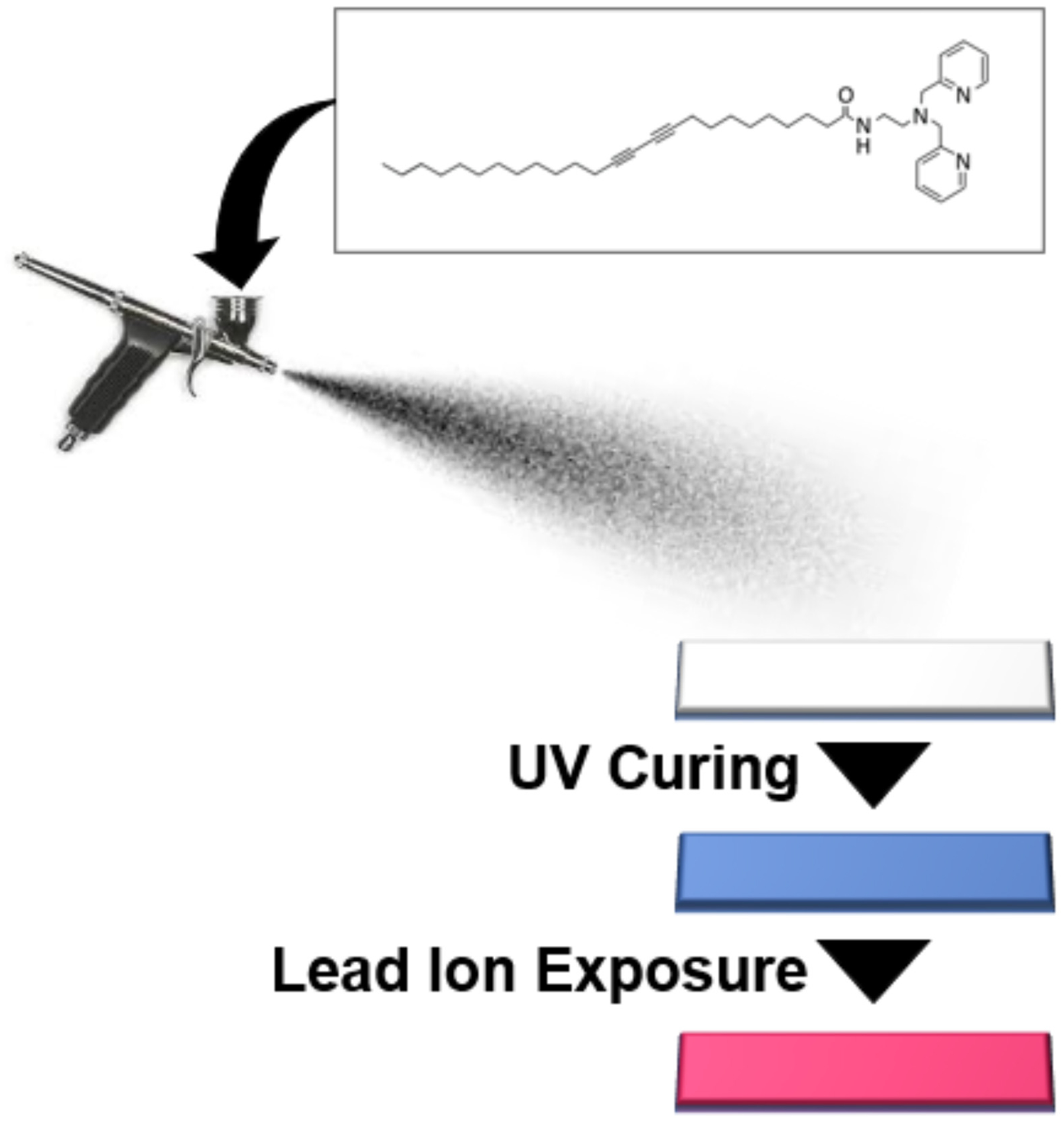
Generalized overview of process for spray-on coating of dipicolylamine-terminating diacetylene-containing amphiphiles onto paper substrate to generate a lead sensor after UV curing and which will exhibit a “turn-on” blue to red color change in the presence of lead ions.

Sprayable sensors have several benefits including the ability to expand chemical sensing to large scale surfaces and to conform to surface contours. Moreover, spray-on sensing materials offer an extremely lightweight coating and are highly flexible, a characteristic that may be particularly appealing for responsive wearable sensing. Spray-on responsive materials represent a new class of sensor that have only recently been explored. Our group, for instance, was the first to demonstrate the use of stand-alone sprayable polydiacetylene formulations for the colorimetric detection of environmental stimuli including chemicals, temperature, or radiation [7]. It is important to distinguish the approach described here from that of manufacturing processes found in many electronic sensors which spray materials as but one step within a multi-step device fabrication scheme. Spray-coating of conducting polymers like PEDOT:PSS have been carried out using stencils in order to deposit patterns as recently demonstrated [8]; however, the spray-on material alone does not provide a fully functional strain sensor, but requires additional components, such as copper wire. Similarly, spray-fabrication of carbon nanotubes has been widely used to create conductive coatings, but these do not function as stand-alone sprayable sensors [9]. In contrast, purely sprayable materials for sensing, on which there is relatively little research, include: fluorescent nanocomposite inks for fingerprint detection [10], FITC-linked aminoethylcellulose particles for pH detection [11,12], porphyrin/coumarin based dyeembedded polymeric nanoparticles for oxygen detection [13], and porphyrin-embedded mesoporous fluorescent particles for pressure detection [14,15], among others. A common feature of such sprayable sensors is the ability of the optical properties to change in the presence of a given stimuli, whether that is a physical or chemical target response. Here we demonstrate that sprayable polydiacetylene-based sensor formulations can provide customizable detection of a target chemical of interest by tailoring the specificity of the pendant side-chain head-group. In this work, we demonstrate a sprayable sensor formulation, which, when coated onto a surface, facilitates a color change for detection of the presence of lead ions in solution. Lead is a toxic heavy metal known to impact brain development, among other health concerns [16–18]. Because unknown exposure through contamination of soil or water could affect public health, development of sensor technologies for the detection of lead ions is of high importance. A variety of methods to modify the head group of pendant side-chains for development of polydiacetylene-based color-responsive materials used in lead detection have been reviewed in literature [19]. In this work, we utilize one such compound that utilizes the dipicolylamine moiety as appended on polydiacetylenes to selectively detect lead ions. Previously, this has been demonstrated in a liposomal formulation [20], but here it is presented as a spray-on sensing material for lead detection. The tridentate dipicolylamine moiety has been well documented for the formation of coordination complexes with various metal ions [21], and for use in a number of different sensing applications, by conjugation with fluorescent reporters [22–28]. In this work, a simple and robust spray-on system can provide detection of the presence of lead ions in a “turn on” color change from blue to red that can be readily observed. As outlined in the schematic below (Figure 1), a formulation comprised of dipicolylamine-terminated diacetylene-containing amphiphiles can be spray-coated onto a substrate (such as paper to provide test strips) and UV-cured to provide a sensor that reveals a visible color change when placed in solutions containing lead ions. While the spray-coating could be applied to most surfaces, here we implemented spraying onto paper substrate to enable a simple color-changing test strip sensor analogous to how ‘litmus paper’ is used for submersion in its testing process. Such disposable sensors can provide accessible information to the wider world at low cost and greater efficiency than biosensors implementing complex measurement techniques [29]. Below, we describe the first spray-on sensor for detection of lead ions, provide details regarding optimization of its color response, and examine its detection limit and selectivity when implemented on a paper substrate. Due to its low cost, this could serve as a model for a widely deployable and disposable sensor.

## 2. Materials and Methods

### 2.1. Synthesis of Dipicolylamine-Terminated Diacetylene-Containing Amphiphile

**Figure 2.**
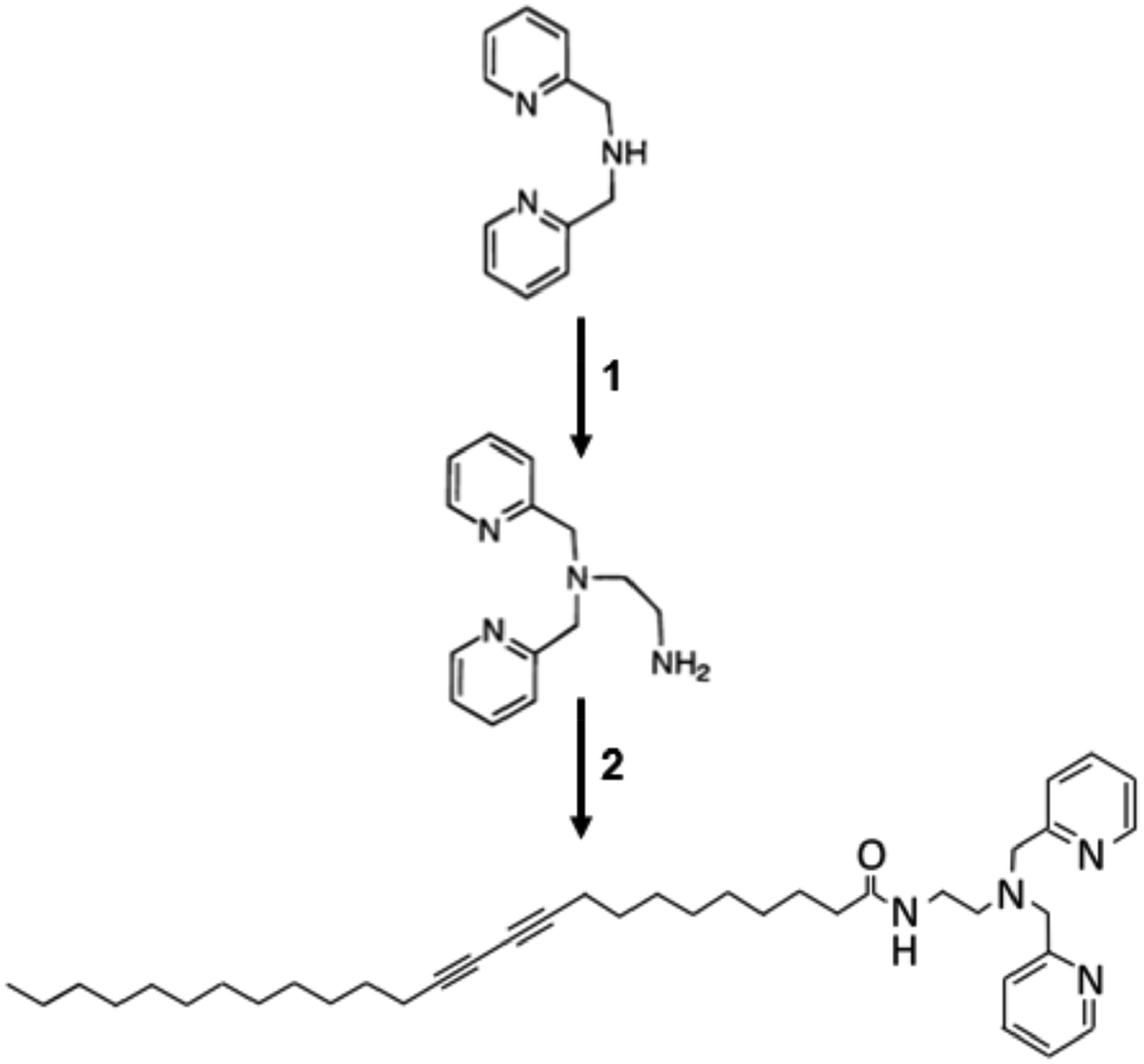
Schematic of two step synthesis of dipicolylamine-terminated diacetylene-containing amphiphiles with formation of (2-aminoethyl)bis(2-pyridylmethyl) amine from di-(2-picolyl)amine in step 1 followed by the formation of N-(2-(bis(2-pyridylmethyl) amino) ethyl) pentacosa-10,12diynamide in step 2.

Synthesis of dipicolylamine-terminated diacetylene-containing amphiphile in this work follows a modification of previous reports and is depicted in Figure 2 [30,31]. Unless otherwise noted, reagents were obtained from Sigma Aldrich Co. (St. Louis, MO, USA). In the first step, synthesis of (2-aminoethyl)bis(2-pyridylmethyl)amine was carried out via initial formation of N-(2-(bis(2-pyridylmethyl)amino)ethyl)-phthalimide which was conducted at 10 mmole scale, specifically, 1.4 mL of triethylamine (Fisher Chemical, Waltham, MA, USA) and 2 g of N-(2-bromoethyl)phthalimide (Acros Organics, Morris Plains, NJ, USA) were added to a mixture containing 1.8 mL of di-(2-picolyl)amine (TCI, Tokyo, Japan) in 20 mL of toluene. This was mixed overnight under nitrogen and underwent reflux with heating at 60 °C. The solvent was then removed using a rotary evaporator (Heidolph Instruments, Schwabach, Germany) until a viscous red oil could be obtained. This red oil was washed with 40 mL of cold water and 40mL of cold diethyl ether, followed by vacuum filtration using a Buchner funnel to obtain a solid product, which was completely dried to obtain the N-(2-(bis(2-pyridylmethyl)amino)ethyl)-phthalimide as a beige solid. Using 1 g of the N-(2-(bis(2-pyridylmethyl)amino)ethyl)-phthalimide, it was dissolved in 20 mL of ethanol and to it added 400 μL of hydrazine monohydrate. This mixture was refluxed under nitrogen at 80 °C for approximately 4 h for the complete formation of a white, gelatinous precipitate. To the mixture, 2 mL of 12 M HCl was added followed by additional heating at 80 °C for 2 h. The resulting suspension was filtered using a Buchner funnel; the filtrate was collected and concentrated under vacuum for removal of ethanol. The collected product was mixed with approximately 12 mL of 1 M NaOH, and the resulting mixture was extracted with diethyl ether. The ether phase was collected and evaporated to obtain the (2-aminoethyl)bis(2-pyridylmethyl)amine in the form of a yellow oil.

In the second step coupling reaction, 500 mg (1.33 mmol) of 10,12-pentacosadiynoic acid monomer (Alfa Aesar, Ward Hill, MA, USA) was dissolved in 20mL of dichloromethane to which 1.175 mL (13.7 mmol) of oxalyl chloride was added dropwise. The reaction proceeded at room temperature with mixing for 4 h, after which, excess solvent was removed by evaporation in a rotary evaporator. The residual product was redissolved in 20 mL of dichloromethane, which was then added dropwise to a solution of (2-aminoethyl)bis(2-pyridylmethyl)amine in dichloromethane, obtained from step 1 as described above. This mixture was stirred overnight at room temperature and the solvent was removed by rotary evaporator. The product was purified by silica gel chromatography using a mobile phase of dichloromethane:methanol:triethylamine at proportion of 100:1:0.1. Pure samples of the dipicolylamine-terminated diacetylene-containing amphiphile (N-(2-(bis(2-pyridylmethyl)amino)ethyl)pentacosa-10,12diynamide) product were collected, analyzed by thin layer chromatography, and pooled. Samples were stored at −20 °C prior to use.

### 2.2. Formulation and Fabrication of Spray-on Lead Sensor Strips

Preparation of the spray-on lead sensor formulations from the amphiphile involved dissolving the amphiphile N-(2-(bis(2-pyridylmethyl) amino) ethyl) pentacosa-10,12diynamide in 100% ethanol at a concentration of 20 mg/mL. The sample were loaded into an airbrush and sprayed directly onto a paper substrate which was used as our testing surface, to enable partial submersion into different ion containing solutions and analysis of the resulting color response. After spray application and prior to solution testing, the substrates that had been sprayed with lead-sensing amphiphile were UV-irradiated with an 8W 254nm UV hand lamp to examine the impact of varying extents of UV curing time.

### 2.3. Testing the Selectivity of the Spray-On Sensor Strips Response to Various Ions

In order to examine the ion selectivity of the spray-on sensor coating, various ion containing solution were prepared. Specifically, 0.1 mM solutions of the following were prepared in 10 mM HEPES (Sigma-Aldrich, H0887): CaCl; CoCl_2_; Co(NO_3_)_2_; FeCl_2_; FeCl_3_; FeSO_4_; KCl; MgCl; MgSO_4_; NaCl; NiSO_4_; Pb(NO_3_)_2_; ZnCl_2_. To test the response, paper substrate was sprayed with the dipicolylamine-terminated diacetylene-containing amphiphile dissolved at 20 mg/mL in ethanol, as described above, and UV-cured with an 8 W 254 nm handlamp for 30 s. The color response was again determined by the distance in (a*, b*) values between submerged and unsubmerged regions of the same strip. Experiments were carried out in triplicate.

### 2.4. Examining the Effect of UV Curing Time on the Spray-On Sensor Response to Lead

To specifically determine the effect of UV curing time on the sensitivity of the coatings, UV irradiation times of 30 s, 1 min, 3 min, and 5 min were implemented. The paper substrates were then cut into strips and partially submerged in 10 mM HEPES buffer containing 1 M Pb(NO_3_)_2_ for 5 min. Pictures of the strips were analyzed in the region exposed to the solution and compared to the unexposed region to determine the color response to the lead stimuli. Specifically, the CIELab a* and b* values were determined for the submerged and unsubmerged areas of the strip. The response to the solution was quantified as the geometric distance between the (a*, b*) ordered pair values in CIELab colorspace for the submerged (exposed) area vs unsubmerged (unexposed) area. Experiments were carried out in triplicate.

### 2.5. Examining the Lead Sensitivity of the Spray-On Sensor Strips

To assess the sensitivity of the color response to lead ion concentrations, serial dilutions of Pb(NO_3_)_2_ were prepared in 10mM HEPES (Sigma-Aldrich, H0887) in the range of 1 M to 10^−8^ M. The pH of the HEPES stock used for this test was 7.5, higher than 7.1, as discussed elsewhere, and was due to lot variability in the commercial HEPES solution (Sigma-Aldrich, H0887), which was reported to be between pH 7.0 to 7.6. As described above, the paper substrate was prepared by spraying with the dipicolylamine-terminated diacetylene-containing amphiphile dissolved at 20 mg/mL in ethanol and with a 30 s UV curing time using an 8 W 254 nm hand lamp. Strips cut from the coated paper were partially submerged for 5 min in the respective dilutions, as well as a control of 10mM HEPES without lead. Experiments were carried out in triplicate and the color response determined based on the CIELab (a*, b*) values as described above.

### 2.6. Examining the Sensitivity of the Spray-On Sensor Strips to pH

To determine if the response of the sensor in the presence of lead was due to changes in pH caused by high concentrations of lead nitrate, we examined both the effect of lead nitrate concentration on pH and the effect of pH on the color of the spray-on sensor strips. For this, 10 mM HEPES buffer and 100 mM HEPES buffer were prepared at pH values of 4.0, 5.2, 6.5, 7.1, 8.2, and 9.7. The spray-on sensor strips, prepared as described above, were partially submerged for 5 min in the 10 mM HEPES buffers of the specified pH values, and the color response was once again determined based on the CIELab (a*, b*) colorspace distance.

## 3. Results

### 3.1. Selectivity in Color Change Response of Spray-On Sensor Strips to Various Ions

**Figure 3.**
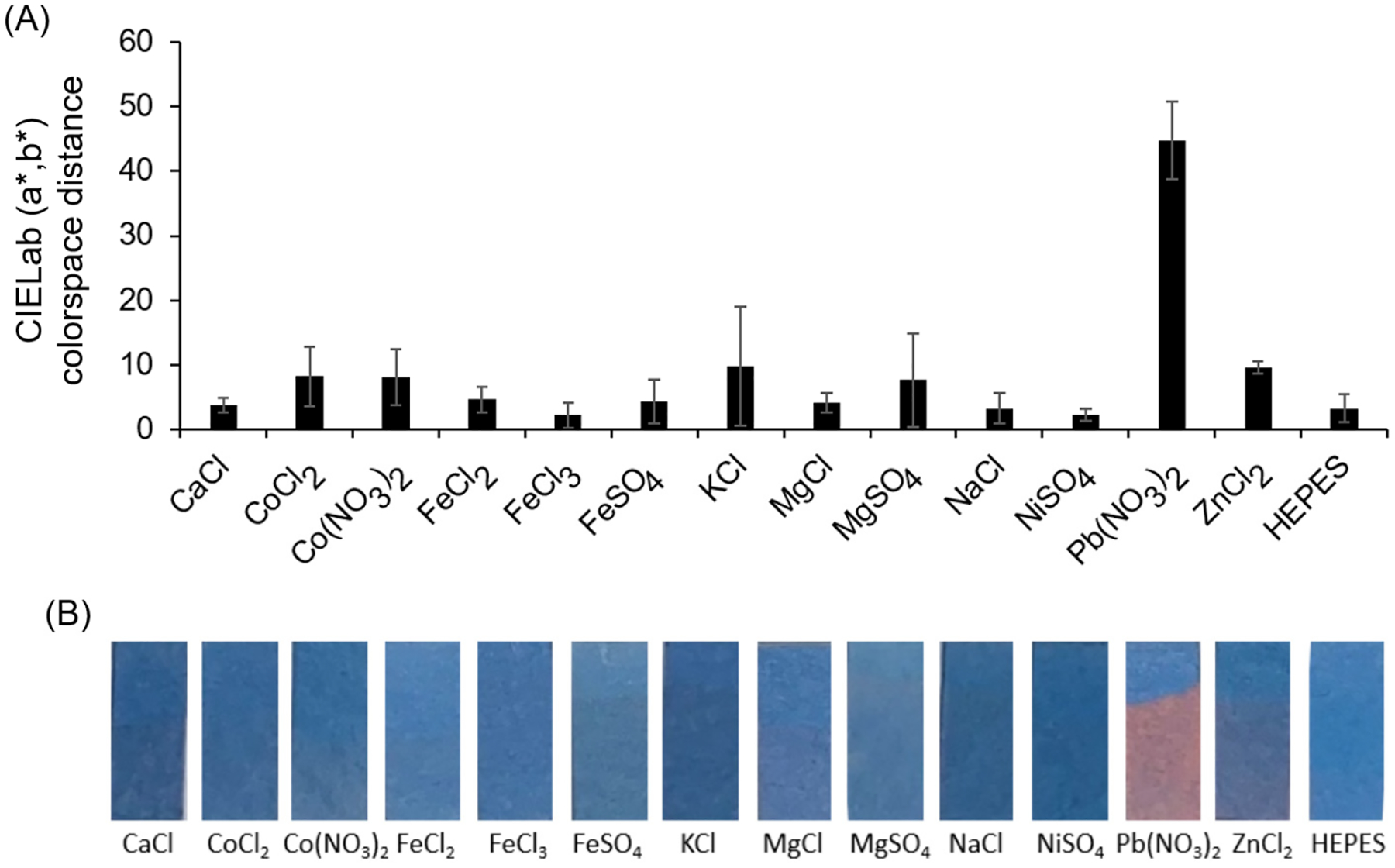
(**A**) Quantification of the color response by calculating the average distance in CIELab (a*, b*) colorspace is provided for each sample tested by comparing the lower (exposed) region to the upper (unexposed) region of the strip. (**B**) Images of the resulting lead sensor strips exposed to 0.1 mM of various ion-containing solutions in 10 mM HEPES (pH 7.1) by partially submerging for 5 min.

Examining the images of the strips sprayed with dipicolylamine-terminated diacetylene-containing amphiphile and partially submerged in solutions containing various ions shown in Figure 3B, the relative color response can be observed. Visually distinct among these is the sample containing Pb(NO_3_)_2_ which produced a clear blue to red color transition. To quantify this response, the average distance in CIELab (a*, b*) colorspace was calculated and revealed as seen in Figure 3A that a response of 10 or less was observed for exposure to any of the anion- and cation-containing samples at 0.1mM, except for the strips exposed to lead ions in the Pb(NO_3_)_2_ samples. The Pb(NO_3_)_2_ response provided an average CIELab (a*, b*) distance of approximately 40, which is at least four times that of the color response to any other ion tested, suggesting this spray-on coating is highly selective for the detection of lead ions in solution.

### 3.2. Effect of UV Curing Time on the Spray-On Sensor Color Response to Lead Ions

**Figure 4.**
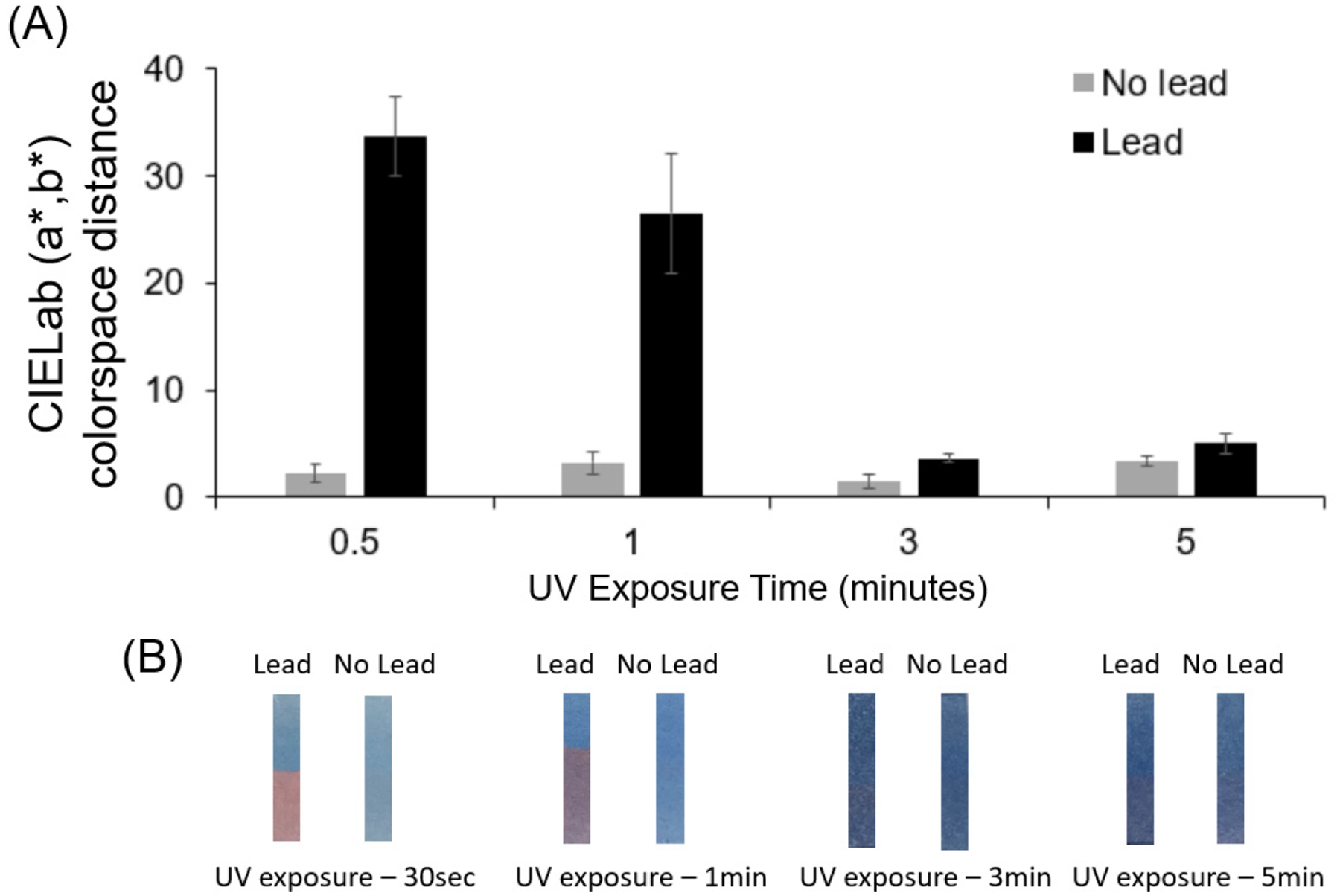
(**A**) CIELab (a*, b*) colorspace distance between submerged region vs unsubmerged region on the image of the sensor test strips with comparison for different UV exposure times. (**B**) Images of sensor strips for different UV exposure times (left)-Lead sensor immersed in 1M lead nitrate for 5 min (right)-Lead sensor immersed in 10mM HEPES for 5 min.

We next examined ways to optimize the lead response of the strips sprayed with dipicolylamine-terminated diacetylene-containing amphiphile. We discovered that the UV curing time had a noticeable impact on the response. As shown in Figure 4, when implementing longer UV curing times by exposure to the 8 W 254 nm handlamp for polymerization of the diacetylene containing amphiphiles, we observed a decrease in the visible color response. While longer UV irradiation times provide a darker initial blue color for the sensor, it does not prove beneficial to use lengthy irradiation as it reduces the color response to lead ions. As seen in Figure 4, the shortest UV irradiation time of 30 s and, to a lesser extent, the 1 min UV irradiation time had clear, visually observable differences in color comparisons between the submerged and unsubmerged regions, using the lead ion-containing solution. In contrast, when UV curing was 3 min or greater, there was no observable difference between lead exposed region of the strips and the unsubmerged region. Quantifying this, we see that at 3 min or greater, the response is similar to the control (an average CIELab (a*, b*) colorspace distance less than 5), which is not visually discernable. In contrast, an average colorspace distance of approximately 35 was observed for the lead response when using the strips produced with a 30 s UV curing time. This short time for UV irradiation provided a desirable response and, hence, was used for the sensitivity assessment.

### 3.3. Sensitivity in Response of Spray-On Sensor Strips to Lead Nitrate

**Figure 5.**
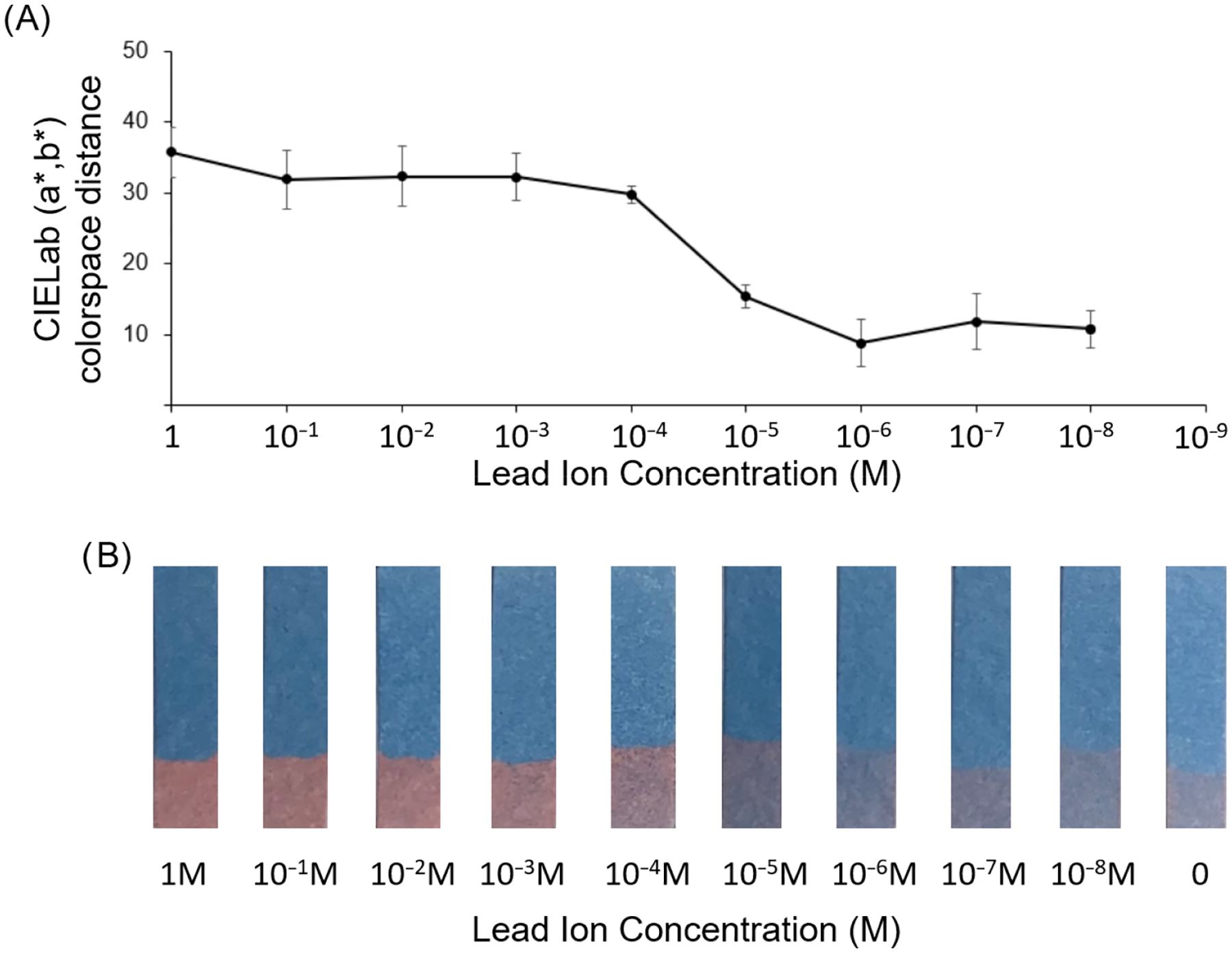
(**A**) CIELab (a*, b*) distance for lead sensor strips exposed to serial dilutions of lead nitrate in 10mM HEPES (pH 7.5). (**B**) Images of test strips after partial submersion in lead nitrate solution of respective concentrations for 5 min.

To examine the concentration-dependent sensitivity in the spray-on sensor to lead ions and determine the detection limit, the color response was quantified across a large range of Pb(NO_3_)_2_ concentrations from 1M down to 10 nM. As seen from Figure 5, the response of the strips sprayed with dipicolylamine-terminated diacetylene-containing amphiphile revealed a concentration-dependent response with, what appears to be, a maximal color response at concentrations of 10 μM and higher. At this high concentration range, a 30 to 35 CIELab (a*, b*) colorspace distance was calculated when comparing the submerged region to the unsubmerged area of the sensor strips. In contrast, a CIELab (a*, b*) distance of 10 or less was found for concentrations of Pb(NO_3_)_2_ at 1 μM and below, which indicates an insufficient color distinction between the submerged and unexposed areas when Pb(NO_3_)_2_ is present at 1 μM or less. The visible detection limit for lead ions is, thus, in the range of 1–10 μM. Due to variability in commercially obtained HEPES (between pH 7.0 to 7.6 for Sigma-Aldrich, H0887), these experiments with pH 7.5 HEPES produced a slightly higher, but visually noticeable, CIELab colorspace distance measurement for the submerged region. This color change is still well below the obvious blue-to-red response seen for 10 μM lead nitrate concentrations above. Interestingly, the maximal color response at 100 μM does not seem to increase much when even higher concentrations are used, suggesting saturation in the response has been reached.

In order to confirm that the color response seen for 10 μM and higher concentrations of lead was attributed to interactions with the dipicolylamine headgroup, we carried out an additional testing of lead ion exposure to spray-on strips using the diacetylene monomer 10,12-pentacosadiynoic acid, which lacks the dipicolylamine head group. The results seen in Figure S1 show no significant change in the CIELab (a*, b*) colorspace distance, as well as no visually detectable color change in response to lead ions for this control test strip. Indeed, this suggests that the detection among the lead sensor strips exposed to increasing concentrations of lead ion, as shown in Figure 5, is caused by interaction with the dipicolylamine headgroup.

### 3.4. Assessing the Role of Sample pH on Color of Spray-On Sensor Strips

**Figure 6.**
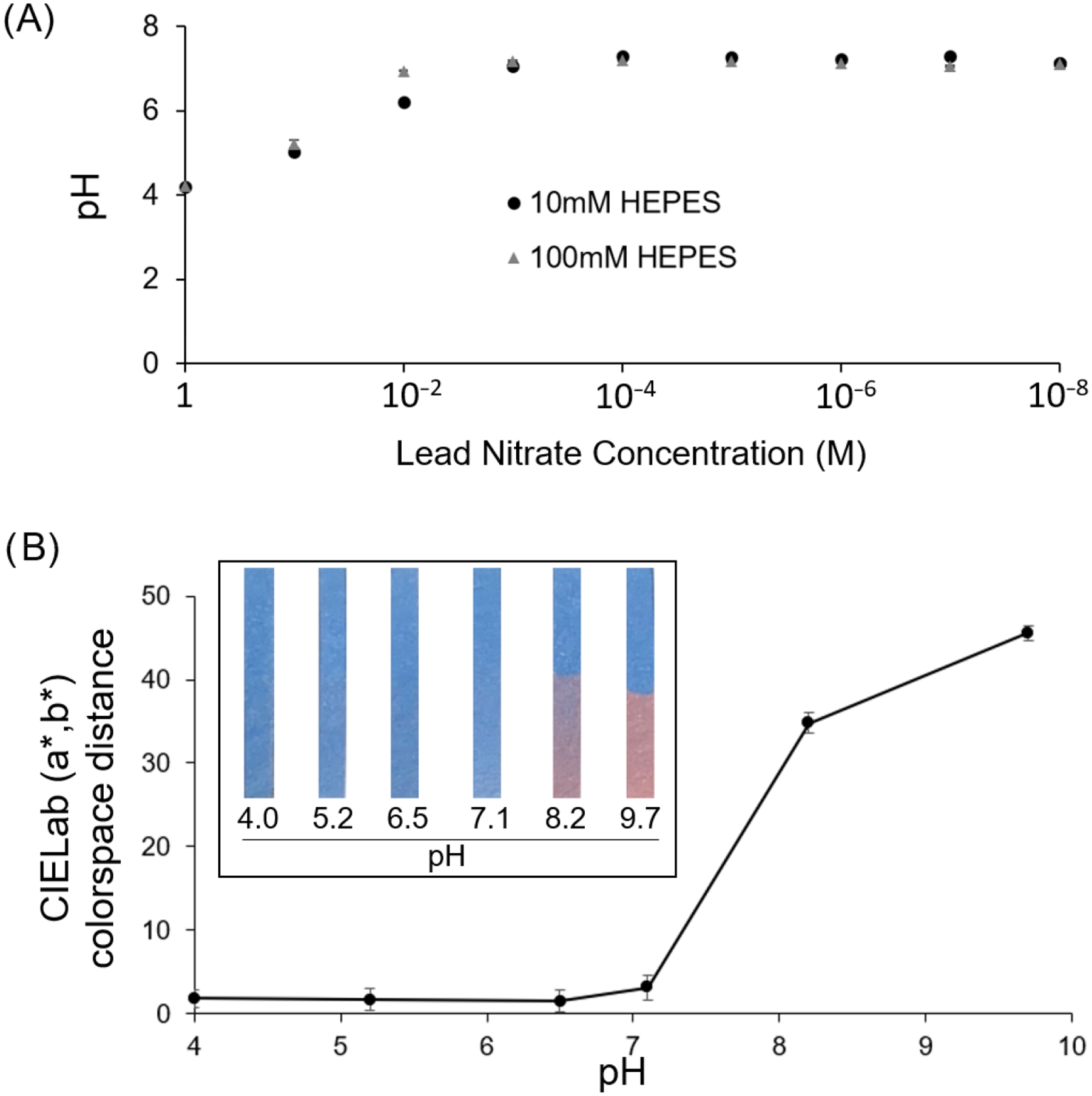
(**A**) Examining the effect of lead nitrate concentration on pH of the carrier buffer (HEPES) revealing that a concentration of 0.1M lead nitrate or higher results in an acidic pH. (**B**) Response of the lead sensor test strips to HEPES buffers of varying pH showing CIELab (a*, b*) distance for lead sensor strips and inset of example images of test strips after partial submersion in HEPES having the respective pH level for 5 min. Results show that the sensor strips have a stable color at low pH (7.1 to 4) and thus would not be impacted by acidic pH resulting from higher lead nitrate concentrations.

In order to first assess if the sensor response observed for lead ions was attributed to pH effects of high lead nitrate concentration, the pH of the HEPES carrier buffer was assessed across the range of 1 M to 10 nM lead nitrate as shown in Figure 6A. A substantial decrease in the pH value was observed at 1 M and 0.1 M lead nitrate concentrations to produce an acidic sample. To next assess the pH response of the spray-on sensor, strips sprayed with dipicolylamine-terminated diacetylene-containing amphiphile were UV-cured for 30 s and partially submerged in 10 mM HEPES buffers having pH values of 4.0, 5.2, 6.5, 7.1, 8.2, and 9.7. As seen in Figure 6B, the sensor strips were not susceptible to color changes due to acidic sample conditions; however, basic condition of pH 8.2 and above caused the sensor to undergo a blue-to-red color change suggesting that the sensors could not be used for detection of lead ions in samples with high (alkaline) pH. Because the lead nitrate at high concentrations results in a low pH (reaching pH 4 for 1M lead nitrate) and does not cause the sample pH to increase, the observed color response of the sensor strips to lead nitrate can be attributed to the lead ions, rather than the observed decrease in pH. In fact, the measured values of the CIELab (a*, b*) colorspace distance were found to be very stable between pH 4 and 7.1, with no significant change across this range.

## 4. Discussion

### 4.1. Summary

Various dipicolylamine-containing sensing systems have been implemented for detection of metal ions, due to the ability of the pyridyl arms of this ligand to bind with Pb^2+^, Fe^3+^, Zn^2+^, and K^+^ [28,31]. Here, we utilize a dipicolylamine-terminated diacetylene-containing amphiphile, which has previously been shown to work in liquid-phase liposomal sensing systems, for the specific detection of lead ions [20,21]. In contrast to prior reports, we demonstrate here for the first time its implementation as a sprayable sensor formulation which can transform a surface into a lead ion detection system capable of a direct “turn-on” visible color change. As shown in this work, we successfully demonstrated implementation of a sprayable lead sensor by deposition onto a paper substrate to create test strips for easily visible lead ion detection. They have a high level of selectivity, demonstrated by a visible color change that only occurs in the presence of Pb^2+^ and good sensitivity, with a detection limit in the range of 1–10 μM. The sensor did not exhibit a significant range of linearity in the response, but rather operated in a binary, “turn-on” mode by undergoing a visible blue to red color change. Future examination of the response at higher resolution, between 1 μM to 10 μM, may show a linear response over this narrow range; nonetheless, enthusiasm is high for the observed “turn on” response to lead ions demonstrated in this spray-on sensor that reliably and specifically provides a binary blue to red color readout. While this sensor may perform best as a binary sensor for lead detection to provide a “No-or-Yes” response corresponding to a blue-red color change, the system could still be implemented in a semi-quantitative manner if utilizing dilutions and assessing the cutoff concentration near 10 μM. For example, semi-quantitative detection to estimate the approximate lead concentration within an unknown sample could be conducted based on a calibration curve, as can be done with other binary sensing systems. In brief, a sample of unknown lead concentration would undergo serial dilutions and a control sample containing 1 M lead ions as the initial standard would undergo the same dilutions. Strips of the lead sensor would be exposed to each of the dilutions of unknown and control samples. The dilution at which a blue to red color change was no longer observed would be designated the lead concentration cutoff, based on the standard calibration with the control sample (such as 1–10 μM in our results). The approximate concentration of lead in the original stock could, thus, be determined within an order of magnitude.

Another notable outcome in this work is the observation that the color response to Pb(NO_3_)_2_ was four times greater than any other anion or cation tested, all of which remained at the threshold of being visually undetectable. It is interesting to see the high degree of selectivity here when compared with reports that dipicolylamine is capable of binding with Pb^2+^, Fe^3+^, Zn^2+^, and K^+^ [20,28,30,31]. While we do see in our study that Zn^2+^ and K^+^ give the next highest color response, they are still at a low CIELab colorspace distance of ~10 which is almost not visible at the 0.1 mM concentration examined. Differences in selectivity reported in literature between fluorescence-based dipicolylamine-containing ligand systems [23–27] and dipicolylamine appended diacetylene amphiphile systems [20,26] are expected to be attributed to differences in the detection mechanisms reported. Specificially, the former are associated with quenching and the latter result from stabilization and rearrangement of head-groups on the pendant side-chains of polydiacetylenes. Reports have shown that coordination of the electron-donating nitrogen of the two pyridyl arms and the central amine of the dipicolylamine to form complexes with metal ions contribute to the binding selectivity [22,24]. The addition of linker segments with an additional amine, such as in the formation of (2-aminoethyl)bis(2-pyridylmethyl)amine, provides a fourth coordination site that can modify relative affinity for metal ions [25,27]. Dipicolylamine derivatives linked via amide bonds to fluorophores [22] or even diacetylene-containing amphiphiles [20] demonstrate utility in the detection of lead ions, wherein the binding mechanisms proceed as described above, through the two pyridyl nitrogens and tertiary nitrogen, but with the added contribution of the fourth coordination site through the amide bond’s carbonyl oxygen. This has also been reported in fluorescently labeled dipicolylamine derivatives, but with different amide tautomer-binding modes contributing to complex formation with Cd^2+^ vs Zn^2+^ ions in aqueous solutions [24]. In lead ion detection with dipicolylamine-terminated diacetylene-containing amphiphile, which possesses this amide linker segment, a prior report of the compound used in this study proposed that the color response of the polydiacetylene material binding lead ions occurs due to interaction between the tridentate dipicolylamine unit on one monomer and the carbonyl oxygen of an adjacent monomer [20]. It has been suggested that this interaction between the head-groups of the adjacent polydiacetylene side-chains causes perturbation in the orientation of the side-chains, distortion of the aligned p-orbitals of the polydiacetylene backbone, shortening of the effective pi-conjugated chain length, and the resulting blue-to-red color change, which are the commonly accepted mechanisms involved in the polydiacetylene-based analyte-binding induced color response [19,26,32,33].

### 4.2. Discussion and Prospects

Interestingly, we discovered that we could manipulate the extent of color response of this spray-on lead sensor by modifying the UV curing time. Specifically, a shorter time of UV irradiation provided a greater color response to the same level of lead ion, as compared to a longer exposure time to UV irradiation. As UV exposure is known to initiate 1,4-addition polymerization of adjacent diacetylene-containing amphiphiles, the optimal response at lower UV curing times suggests that an incomplete polymerization may more readily allow lead-induced conformational changes in the conjugated pi backbone, as compared to highly polymerized surface coatings that may restrain movement. Because these rearrangements in the pi backbone correspond to a higher degree of observable shift in absorbance that yields the blue to red color transition, it is clear that stable detection requires a sufficient amount of response to be observable from the outermost layer, which is the only portion of the spray-on sensor coating in contact with the sample. The decrease in observable color change in response to lead when using longer exposure times of UV curing could potentially be the result of blue-phase material forming deeper within the coating, not at the outermost layer, and hence, cannot interact with the sample. In this case, a relatively smaller amount of the visually observable sensing material (specifically, the outermost material capable of interacting with sample) would be exposed to lead ions in the sample. As a result, a smaller subset of the observed blue coating could be available to undergo a change to the red phase making the visual response indistinguishable due to a larger proportion of unexposed blue phase background color from deeper within the coating.

Another interesting feature of the lead sensor was the observed large color response at 0.1 mM of lead ion that remained consistent for even higher concentrations of lead suggested that some degree of saturation may have been reached. It remains unclear if this saturation point can be tuned by utilizing a mixture of diacetylene-containing amphiphile in which only a fraction of the head-groups contain dipicolylamine; however, this could suggest that alternative formulations may be possible to develop that enable the “turn-on” response to occur at distinct, customizable concentrations. As characterization of this new class of spray-on sensor is in its early stages, further work exploring this class of stimuli-responsive sprayable coatings is warranted. We hope that future spray-on formulations of chemically responsive materials can expand chemical sensing to large scale surfaces which could enable inexpensive and widely deployable sensing.

## Supplementary Material

Supplementary Data
