# Supplementary material for: Spray-On Colorimetric Sensors for Distinguishing the Presence of Lead Ions
Source: Chemosensors (Basel). Author manuscript; Available in PMC 2024 Mar 8. (PMC10923167; doi:10.3390/chemosensors11060327)
Supplement: Supplementary Data [file NIHMS1971394-supplement-Supplementary_Data.pdf]

Supplementary Data

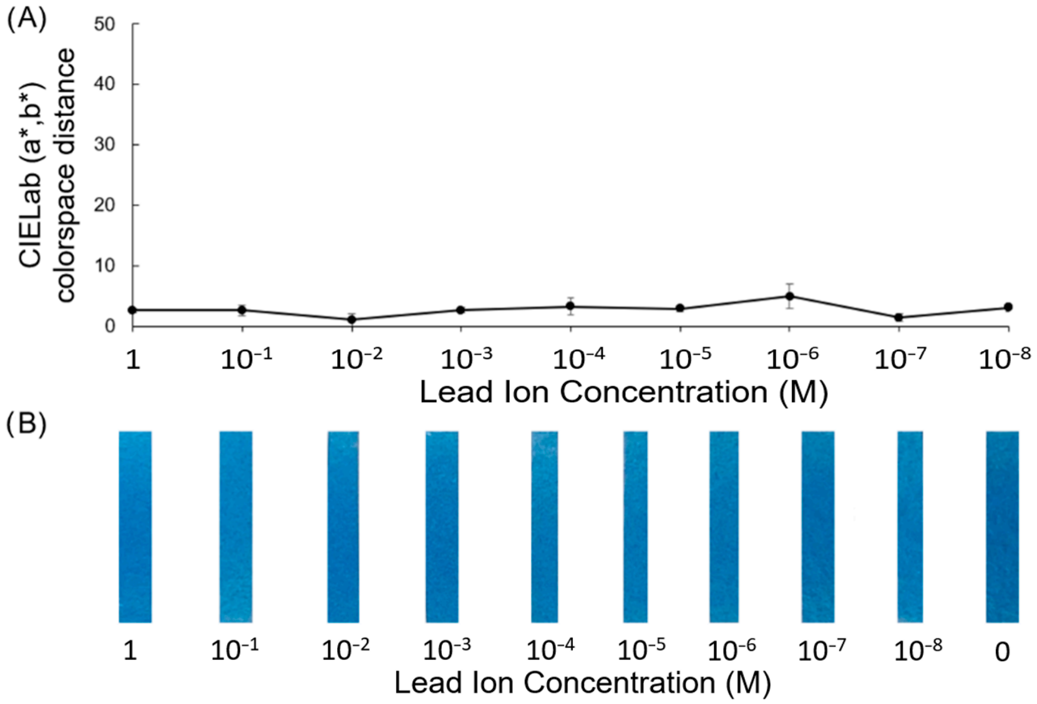

Figure S1. A) CIELab (a*, b*) distance for strips made by spray-on of PCDA as a comparative group (and not the dipicolylamine-conjugated lead sensor) exposed to serial dilutions of lead nitrate. B) Images of PCDA strips after partial submersion in lead nitrate solution of respective concentrations for 5 minutes showed no observable color change.

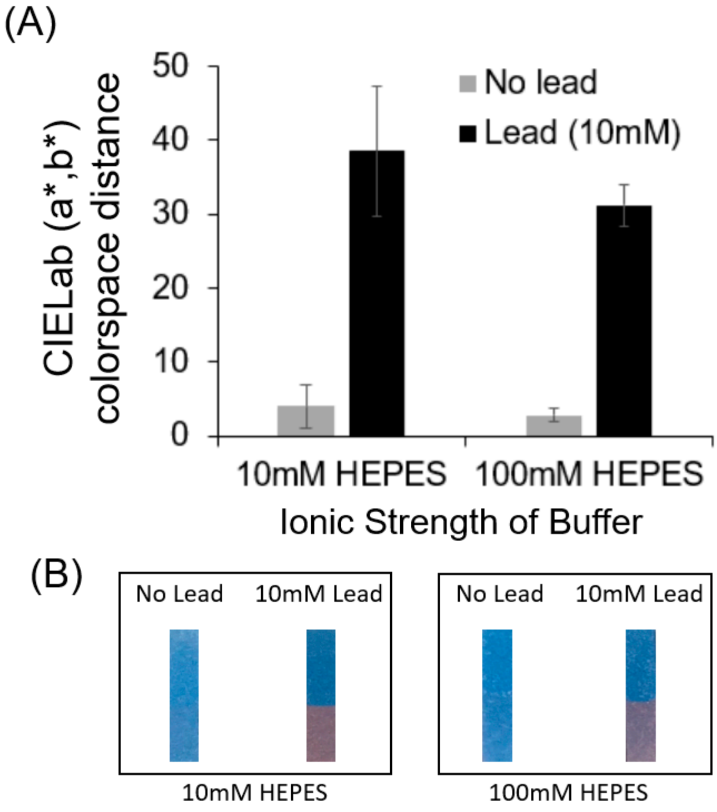

Figure S2. A) Examining the effect of ionic strength of the carrier buffer (HEPES) on the response of the test strips to lead nitrate. CIELab (a*, b*) distance for lead sensor strips exposed to 10mM of lead nitrate or no lead nitrate using a buffer of either 10mM or 100mM HEPES. B) Images of test strips after partial submersion in lead nitrate solution of respective HEPES concentrations for 5 minutes showing that both 10mM HEPES and 100 mM HEPES had similar blue-to-red color responses to lead ions.

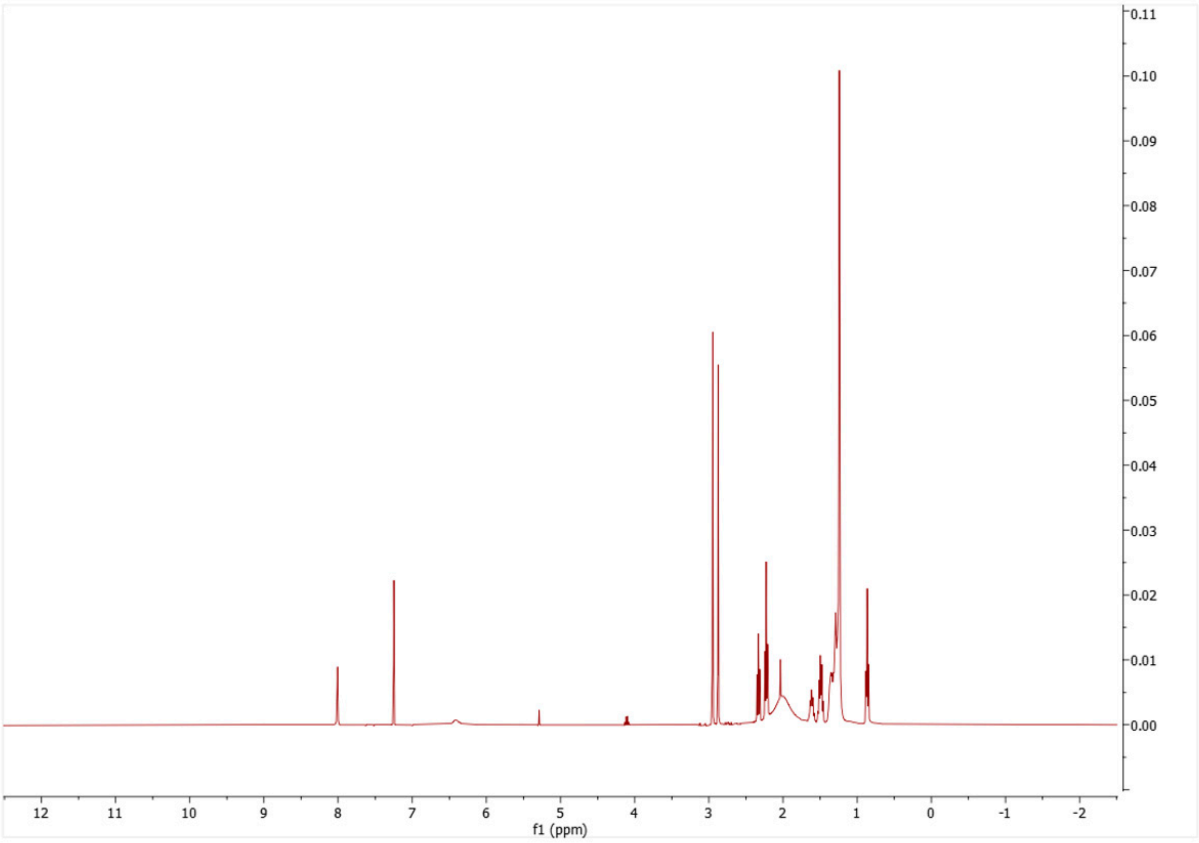

Figure S3. ^{1}H NMR spectra of product N-(2-(bis(2-pyridylmethyl) amino) ethyl) pentacosa-10,12diynamide (400 MHz, $CDCl_3$).
